# A global analysis of conservative and non-conservative mutations in SARS-CoV-2 detected in the first year of the COVID-19 world-wide diffusion

**DOI:** 10.1038/s41598-021-04147-1

**Published:** 2021-12-30

**Authors:** Nicole Balasco, Gianluca Damaggio, Luciana Esposito, Flavia Villani, Rita Berisio, Vincenza Colonna, Luigi Vitagliano

**Affiliations:** 1 Institute of Biostructures and Bioimaging, National Research Council (CNR), Naples, Italy; 2 Institute of Genetics and Biophysics, National Research Council (CNR), Naples, Italy

**Keywords:** Computational biology and bioinformatics, Structural biology

## Abstract

The ability of SARS-CoV-2 to rapidly mutate represents a remarkable complicancy. Quantitative evaluations of the effects that these mutations have on the virus structure/function is of great relevance and the availability of a large number of SARS-CoV-2 sequences since the early phases of the pandemic represents a unique opportunity to follow the adaptation of the virus to humans. Here, we evaluated the SARS-CoV-2 amino acid mutations and their progression by analyzing publicly available viral genomes at three stages of the pandemic (2020 March 15th and October 7th, 2021 February 7th). Mutations were classified in conservative and non-conservative based on the probability to be accepted during the evolution according to the Point Accepted Mutation substitution matrices and on the similarity of the encoding codons. We found that the most frequent substitutions are T > I, L > F, and A > V and we observe accumulation of hydrophobic residues. These findings are consistent among the three stages analyzed. We also found that non-conservative mutations are less frequent than conservative ones. This finding may be ascribed to a progressive adaptation of the virus to the host. In conclusion, the present study provides indications of the early evolution of the virus and tools for the global and genome-specific evaluation of the possible impact of mutations on the structure/function of SARS-CoV-2 variants.

## Introduction

In the last months of 2019, a novel and severe acute respiratory syndrome emerged in the Chinese city of Wuhan. Within a few weeks, this local disease spread worldwide leading the World Health Organization to declare the outbreak “a public health emergency of international concern” (January 30th 2020). The causative agent of this disease was identified on December 31st in a novel coronavirus (Severe Acute Respiratory Syndrome Coronavirus 2—SARS-CoV-2) whose first genome sequencing was reported in mid-January 2020 (GISAID accession ID: EPI_ISL_402124) (https://www.gisaid.org/)^1^. Despite the enormous efforts made globally, the development of effective therapeutic or preventive approaches for this disease is still an ongoing process. Among others, the ability of SARS-CoV-2 to mutate rapidly represents a remarkable complicacy. SARS-CoV-2 is a Baltimore class IV^2^ positive-sense single-stranded RNA virus and is a member of the subgenus Sarbecovirus (beta-CoV lineage B)^3^. Its RNA sequence contains approximately 30,000 bases (GISAID; https://www.epicov.org)^1,4^ that encode 28 distinct proteins. Since the publication of the first SARS-CoV-2 genome, a remarkable number of variants have been daily characterized. This provides a unique opportunity to monitor the evolution of the mutations during the process of the virus adaptation to the host in a sort of evolution in action. As typically observed in viruses^5^, SARS-CoV-2 presents a remarkable propensity to mutate. The estimated mutation rate of SARS-CoV-2 is about 9.8 × 10^–4^ substitutions *per* site *per* year^6^. Although only indirectly related to the mutation rates, several studies analyzed the distribution and frequencies of the observed SARS-CoV-2 mutations. These analyses have been conducted on the entire genome or on specific proteins considered to be crucial for the development of effective therapeutics interventions^6–12^. In the present paper, we evaluated the SARS-CoV-2 amino acid (AA) mutations at three stages of the pandemic: 2020 March 15th, 2020 October 7th, and 2021 February 7th. In particular, we classified the mutations in conservative and non-conservative ones based on the probability to be accepted during the evolution according to the Point Accepted Mutation substitution matrices^13^ and on the similarity of the encoding codons^14^. The comparative analysis of mutations detected at these three stages of the pandemic unravels significant analogies despite the huge difference in their overall content. The present study provides some indications of the early evolution of the virus and useful tools for the global and genome-specific evaluation of the impact that mutations could have on the structure/function of SARS-CoV-2 variants that emerged or will emerge in the pandemic.

## Results

To monitor the evolution of the SARS-CoV-2 virus in the first months of the pandemic we evaluated the amino acid (AA) substitutions retrieved from the GISAID database (https://www.gisaid.org/)^1^ at 2020 March 15th (DataMar20—Supplementary Table S1), 2020 October 7th (DataOct20—Supplementary Table S2), and 2021 February 7th (DataFeb21—Supplementary Table S3). Using the sequence of the Wuhan genome as reference (GISAID accession ID: EPI_ISL_402124) we considered mutations occurring in the same position of each viral protein only once even if present in different genomes. While this choice does not provide information on homoplastic mutations, the occurrence of this kind of mutations has proved to be minimal compared to the global number of substitutions detected in the virus genomes^6,12^.

### Definition of conservative and non-conservative mutations

The AA substitutions were classified according to two criteria. First, we considered the Point Accepted Mutation (PAM) score, i.e. the likelihood that an AA substitution is accepted by natural selection based on the probability of finding the same mutation in highly homologous proteins^13^. In particular, after considering the very low percentage of mutated AAs per SARS-CoV-2 genome, we referred to the mutation probability matrix of PAM1 that reports the probability of a specific AA replacement in sequences that are 1% different^13^. The PAM values used in this work are the probabilities reported in the PAM1 matrix multiplied by 10,000 (Supplementary Table S4). Second, we considered the number of base changes required at codon level to generate the AA replacement. It has been recently reported that out of 380 possible AA substitutions, some (150) may occur with a single base change in the codon whereas the others (230) require more than one base change in the genetic code to happen^2^ (Table 1).

**Table 1:** Grouping of the AA substitution types according to the Point Accepted Mutation (PAM) score and the number of base changes required at the codon level. Theoretical values and the number of AA substitution types detected in DataMar20, DataOct20, and DataFeb21 are also reported.

	PAM 0–12	PAM > 12	Overall
**Theoretical**			
1 base change	107	43	150
> 1 base change	230	–	230
Total	337	43	380
**DataMar20**			
1 base change	70	37	107
> 1 base change	9	–	9
Total	79	37	116
**DataOct20**			
1 base change	107	43	150
> 1 base change	228	–	228
Total	335	43	378
**DataFeb21**			
1 base change	107	43	150
> 1 base change	230	–	230
Total	337	43	380

**Figure 1:**
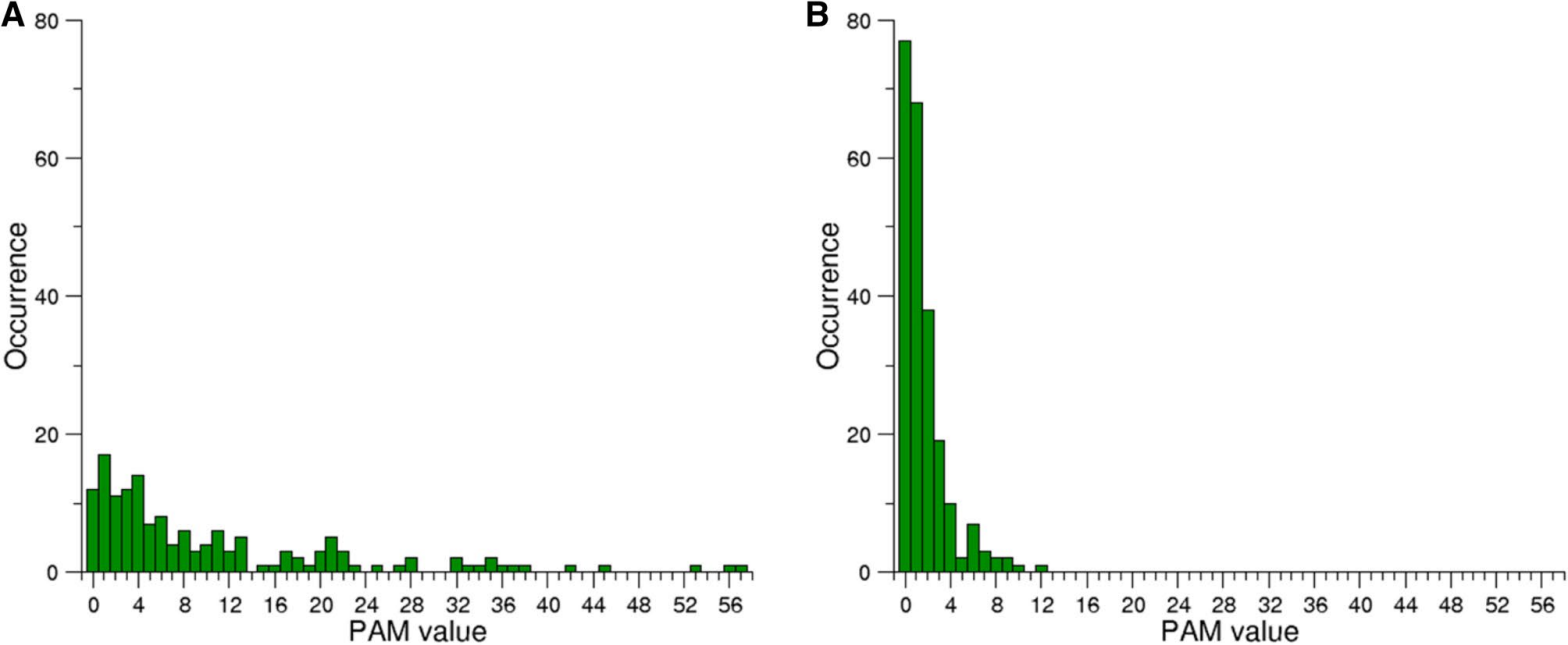
Frequency of the 380 amino acid substitution types that can either occur with a single base change (**A**) or require more than one base change (**B**) as function of the PAM value. The maximum PAM value observed in substitutions that require more than one base change is 12.

We evaluated the interplay between these two criteria by separately plotting the PAM values of these two classes of mutations (Fig. 1). A comparative analysis of Fig. 1A,B clearly indicates that all mutations having PAM values larger than 12 can occur with a single base substitution. On the other hand, we observed that the PAM range of the substitutions requiring more than one change is 0–12 (Fig. 1B). Therefore, based on the fact that high PAM values are associated with changes between AAs that present minimal differences in their chemico-physical properties, and based on the evidence that the maximum PAM value in substitutions that require more than one base change is 12, we define as non-conservative and conservative the mutations with PAM values falling in the range 0–12 and > 12, respectively.

### Analysis of the AA mutations detected at March 2020

The analysis of the mutations occurring in the 581 SARS-CoV-2 genomes deposited in the GISAID database up to 2020 March 15th unravels that 508 of them (87.4%) contain at least one AA substitution compared to Wuhan reference genome. Notably, six of these genomes present more than ten amino-acid mutations, with one (GISAID accession ID: EPI_ISL_406592) having 18 replacements (Supplementary Fig. S1). The inspection of these sequences led to the identification of 404 AA substitutions (DataMar20—Supplementary Table S1). Among these, 395 require a single base change and nine (Y > E, Y > I, W > Y, V > Q, T > F, S > M, L > K, F > R, and A > Q) require more than one change. These nine mutations occur only once and present PAM values that fall in the range 1–3, with seven of them having PAM = 1.

**Figure 2:**
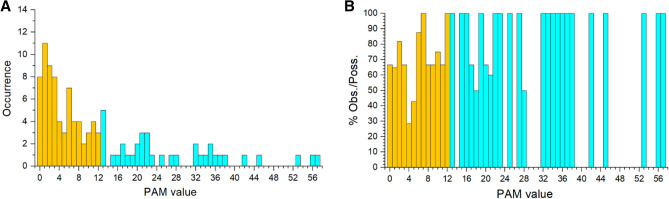
(**A**) Frequency of the 107 types of amino acid replacements that can occur with a single base change detected in the DataMar20 dataset according to their PAM value. In orange substitutions classified as non-conservative, in cyan conservative ones. The overall higher number of non-conservative compared to conservative mutations observed is due to the fact that 337 out of the 380 substitution types have PAM in the range 0–12 and therefore classify as non-conservative. (**B**) Frequency of observed over possible substitutions within PAM values.

In the ensemble of 404 AA substitutions we identified 116 types of AA replacements out of the 380 possible ones (Table 1), i.e. only ~ 31% of the possible substitutions had occurred. Of these, 107 correspond to replacements that can occur with a single base change, i.e. the ~ 71% of the possible substitutions occurring with a single change (150). The distribution of these 107 AA substitution types as a function of the PAM value is reported in Fig. 2A. As most of the substitution types have rather low PAM values (337 out of 380 have PAM < 12, Table 1 and Supplementary Table S4), we plot the percentage of the observed over the possible substitutions within each PAM value. Figure 2B shows that on this relative scale the conservative mutations are almost all realized (37 out of 43; 86.0%) compared to non-conservative ones (70 out of 107; 65.4%), which present a significant number of missing substitution types (Table 1).

**Figure 3:**
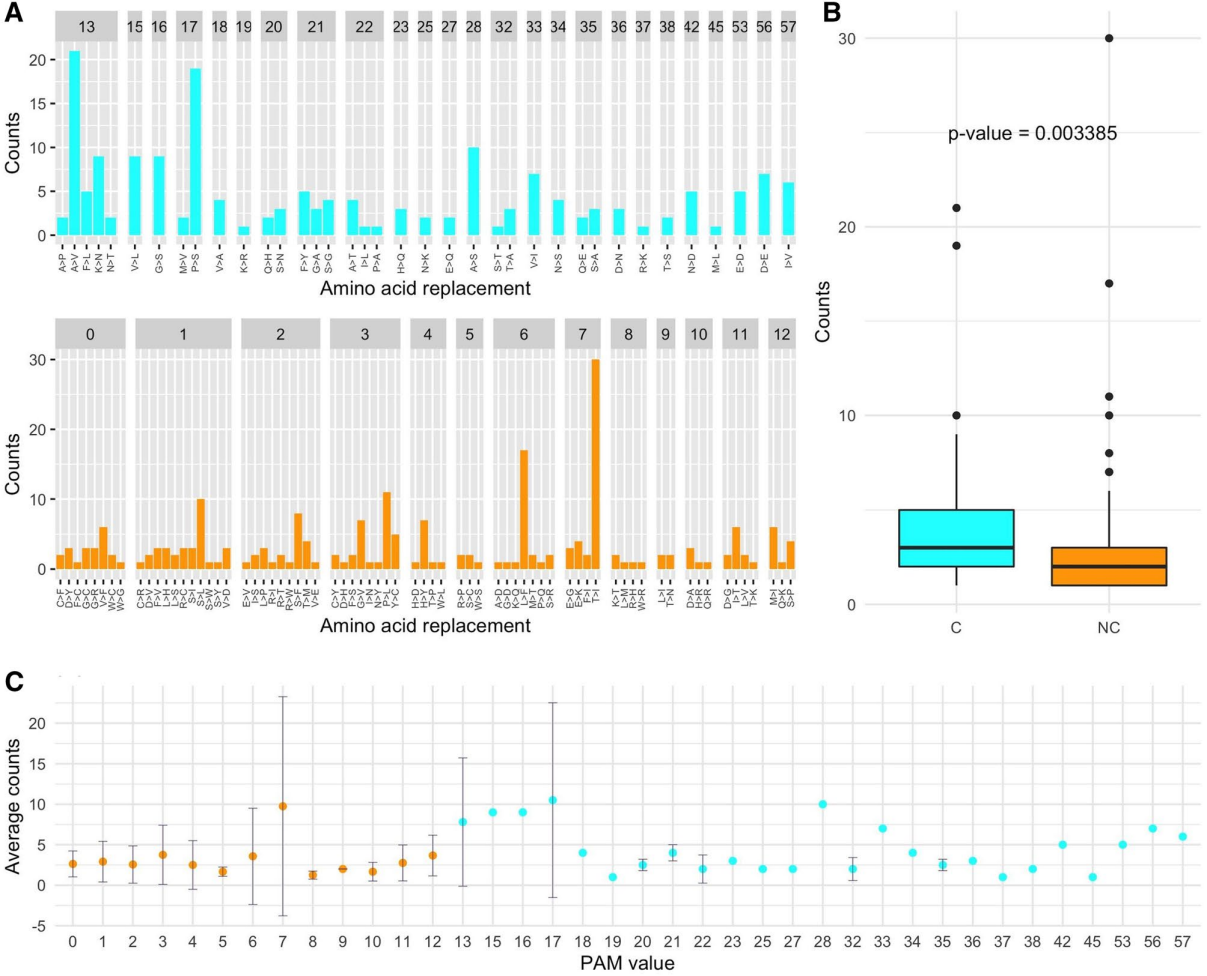
(**A**) Counts of the observed mutations (non-conservative in orange and conservative in cyan) detected in the DataMar20 dataset grouped by PAM value. (**B**) Boxplot of the number of occurrences *per* substitution types. (**C**) Average and standard deviation (bars) of the number of occurrences within PAM values.

Since the 395 AA substitutions that take place within a single base change correspond to 107 substitutions types, on average each substitution type is found 3.7 times (395/107). As shown in Fig. 3A, the observed substitution types have rather different frequencies. Although the most frequent replacement is T > I that is observed 30 times, we observe that conservative substitutions present a significantly higher average number of occurrences compared to the non-conservative (Fig. 3B, Wilcox-test p-value = 0.003).

When considering average occurrences *per* PAM value (Fig. 3C), we observed that some values (6, 7, 13, and 17) have very large standard deviations. This finding suggests that for these values, outliers, i.e. AA substitutions with enhanced frequencies compared to the others sharing the same PAM value, might be present. The inspection of Fig. 3A corroborates this observation as the PAM values of 6, 7, 13, and 17 contain the very frequent substitutions L > F (17 times), T > I (30 times), A > V (21 times), and P > S (19 times), respectively (Supplementary Table S5). Notably, three (L > F, T > I, and A > V) of these most frequent substitutions led to an increase of hydrophobicity (Supplementary Table S6).

**Figure 4:**
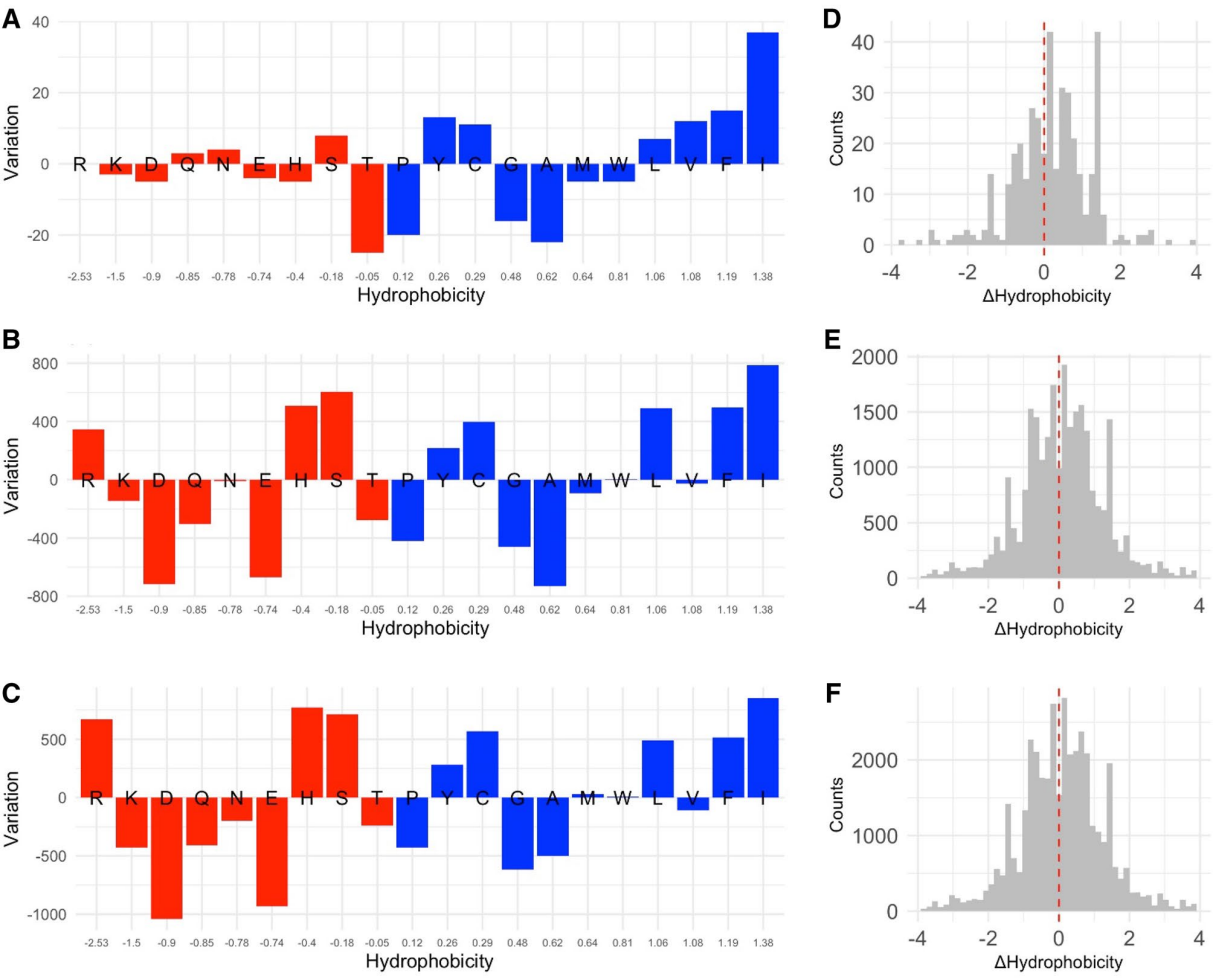
Variation of the total content in amino acids as consequence of the mutations stratified by hydrophobicity in DataMar20 (**A**), DataOct20 (**B**), and DataFeb21 (**C**) datasets. For each amino acid, the enrichment/depletion in counts of mutated versus original residues is reported. Residues with negative or positive hydrophobicity are colored in red and blue, respectively. Distributions of the ΔHydrophobicity for mutations in DataMar20 (**D**), DataOct20 (**E**), and DataFeb21 (**F**) datasets.

To further investigate this aspect, for each AA we estimated its enrichment/depletion in counts of mutated versus original residues. In Fig. 4A we show a trend of enrichment of hydrophobic residues and depletion of the hydrophilic ones that corroborates previous observations. This trend is further confirmed when considering the differences in hydrophobicity (ΔHydrophobicity) between mutated and original residues (Fig. 4D), as the ΔHydrophobicity averaged over all the 404 observed mutations is slightly positive (0.18 ± 1.04).

The classification of the observed AA substitutions following the PAM values was also used to assign a genome divergence index (GDI) to a certain genome with respect to the reference one by considering all the individual AA substitutions present in that genome. Each mutation contributes to the score differently depending on its PAM value (see “Methods” for details). The GDI values calculated for the genomes deposited in the GISAID database up to 2020 March 15th presenting the highest number of mutations is reported in Supplementary Table S7. Although these GDI values are still dominated by the number of the mutations *per* genome, this parameter differentiates genomes having the same number of mutations.

### Analysis of the AA mutations detected at October 2020

**Figure 5:**
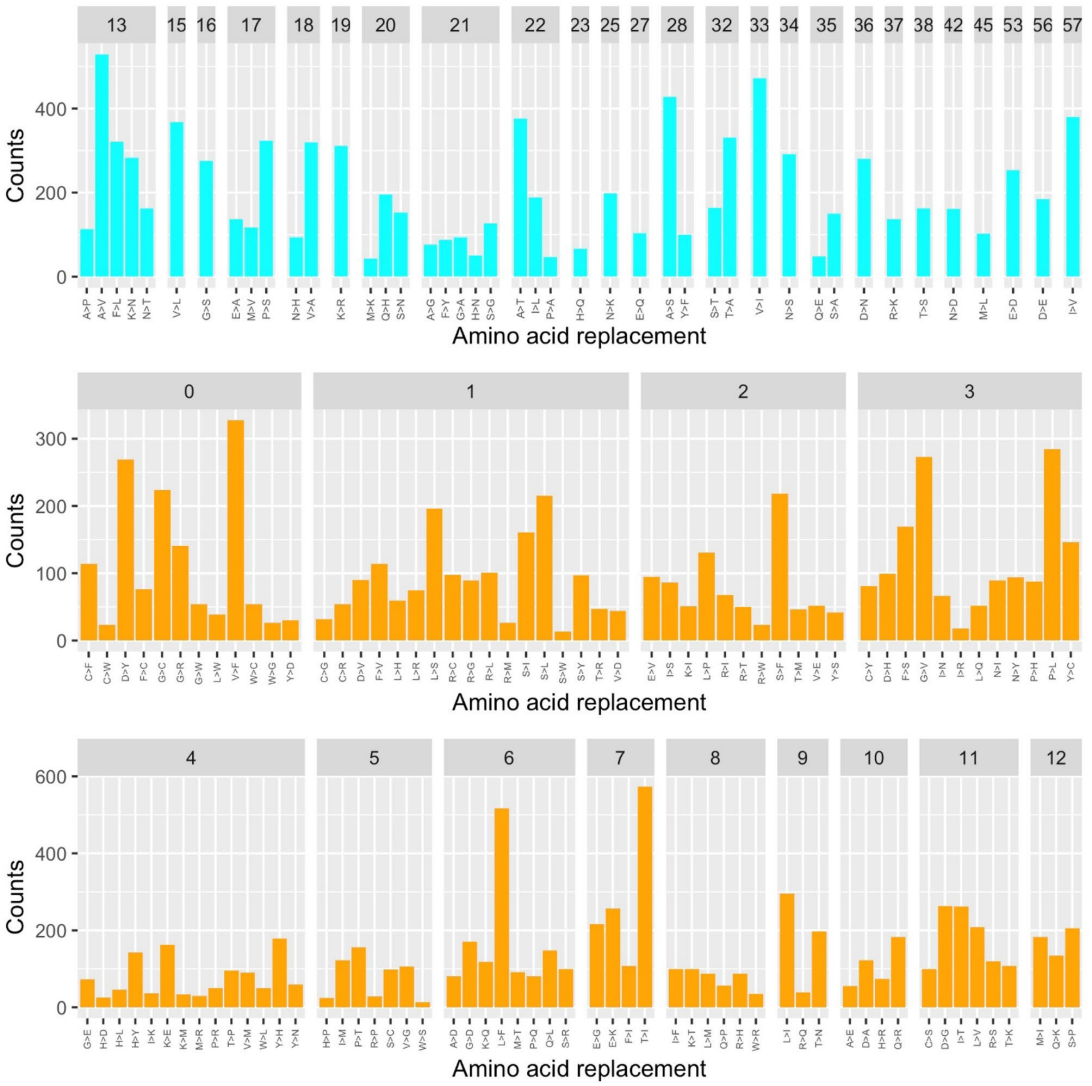
Frequencies of the observed mutations that can occur with a single base change grouped in conservative (cyan) and non-conservative (orange) types detected in the DataOct20 dataset.

**Figure 6:**
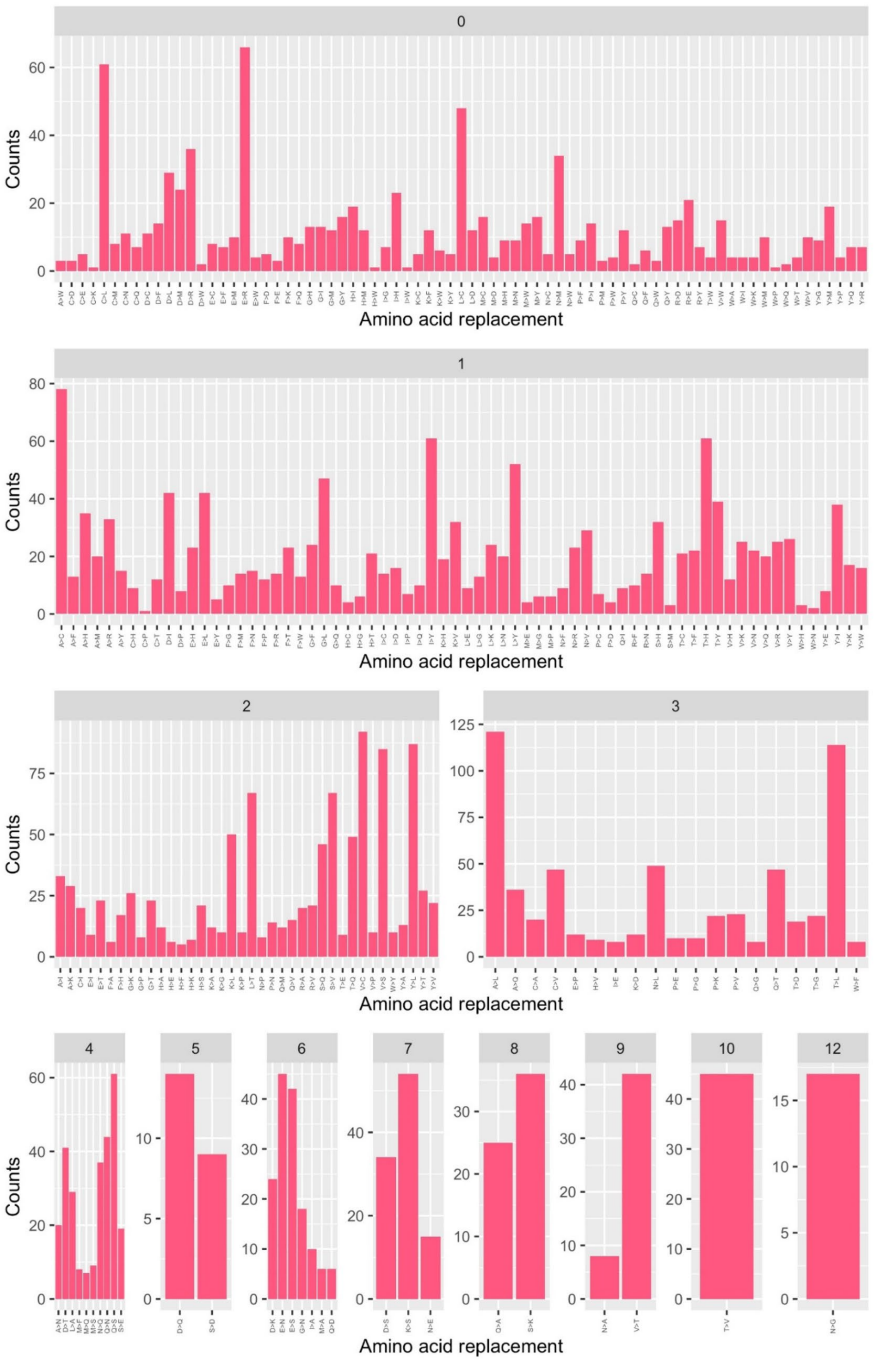
Frequencies of the observed mutations that require more than one base change detected in the DataOct20 dataset.

We compared the trends observed in the DataMar20 dataset with those detected in the dataset DataOct20 (Supplementary Table S2) that contains a much larger number of mutations (25,634) identified in 135,404 genomes. In contrast to DataMar20, almost all possible AA substitutions are present in DataOct20 (378 out of 380) (Table 1). The two missing ones (W > D and W > E) are among the most non-conservative substitutions as they require more than one base change and present a PAM value of zero (Supplementary Table S4). As expected mutations that can occur through a single base change (Fig. 5) present frequencies that are much higher than those requiring multiple base changes (Fig. 6).

**Figure 7:**
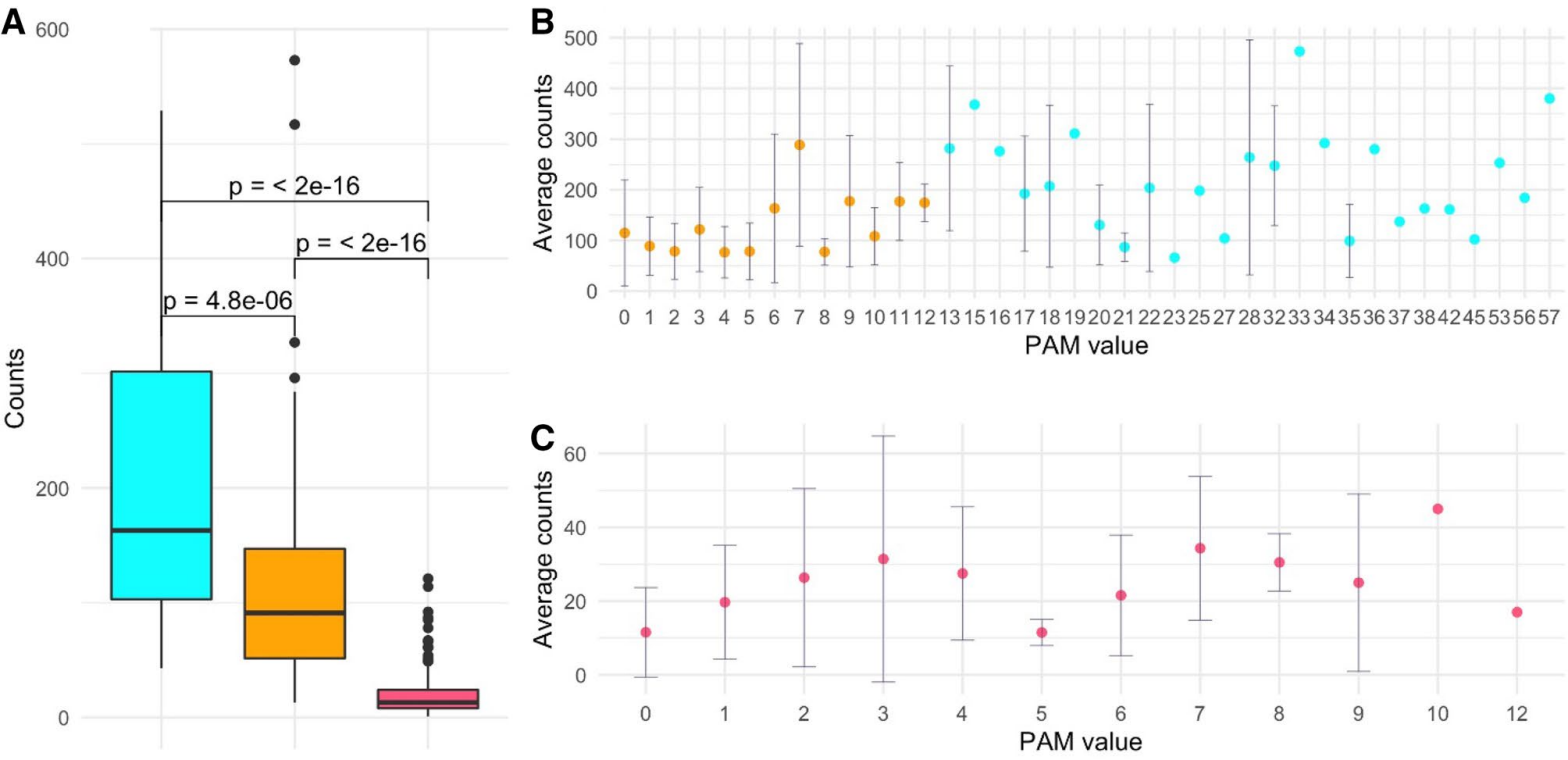
(**A**) Boxplot of the number of occurrences *per* substitution types stratified in conservative (cyan) and non-conservative (orange) types that can occur with a single base change and types that require more than one change (magenta). Average values with standard deviation (bars) of the number of occurrences within PAM values detected in the DataOct20 dataset: (**B**) substitutions that can occur with one base change grouped in non-conservative (orange) and conservative (cyan) and (**C**) substitutions requiring more than 1 base change (magenta).

When analyzing the frequency of the 378 substitution types stratified by PAM values and number of changes required, we observe that conservative mutations are more frequent compared to non-conservative ones (Figs. 5 and 6). Indeed, for mutations occurring with a single base change, the quantitative comparison of the frequencies of the conservative and non-conservative mutations (Fig. 7A) using the Wilcox-test provides a p-value of 4.8 × 10^–6^. As expected, the mutations requiring more than one base change, which are all non-conservative, present significantly lower frequencies compared to the non-conservative mutations occurring in a single base change (Fig. 7A, Wilcox-test p-value < 2 × 10^–16^).

When considering the average occurrences of mutations *per* PAM value, we observe that conservative mutations generally exhibit larger frequencies than non-conservative and that some values present large standard deviations that may be indicative of the presence of outliers (Fig. 7B,C). Similarly to what observed in DataMar20, also in DataOct20 the most frequent replacement is T > I (573 times), followed by A > V (529 times), L > F (517 times), and V > I (473 times) (Fig. 5 and Supplementary Table S5). It is worth mentioning that, despite the general trend outlined above, two of these highly occurring mutations (T > I PAM = 7 and L > F PAM = 6) are non-conservative substitutions. The inspection of occurrences of the mutations requiring more than one base change indicates that A > L (121 times) and T > L (114 times) are the most frequent ones (Fig. 6). Collectively these findings indicate that most frequent substitutions led to an increase of the hydrophobicity, independently of the number of codon base changes required for the mutation.

This is also evident from the analysis of enrichment/depletion in counts of mutated versus original residues as shown by the consistent increase in the two most hydrophobic residues, F and I and the decrease of hydrophilic residues (Fig. 4B). Among hydrophilic residues the arginine (R) is an exception being significantly enriched. This may be ascribed to the complicated hydrophobic/hydrophilic behavior of this residue that presents both a charged group (guanidinium) and an aliphatic chain extending from the C^α^ to the C^δ^ atoms.

As a consequence of these changes, the distribution of the ΔHydrophobicity values is slightly shifted toward an increased hydrophobicity (mean ΔHydrophobicity 0.08 ± 1.16) (Fig. 4E). The large number of mutations contained in DataOct20 allowed the analysis of the ΔHydrophobicity also for the individual viral proteins. This analysis indicates that the rise in hydrophobicity is not uniform but rather driven by some proteins such as the protein N that exhibits the highest value (Supplementary Fig. S2 and Table S8).

Finally, we evaluated the GDI index for the sixty most mutated AA sequences. Although in some cases the index provides different values for genomes with the same number of mutations, the total number of substitutions dominates its value (Supplementary Table S9). This is due to the fact that all of these genomes include many mutations with low PAM values.

### Analysis of the AA mutations detected at February 2021

**Figure 8:**
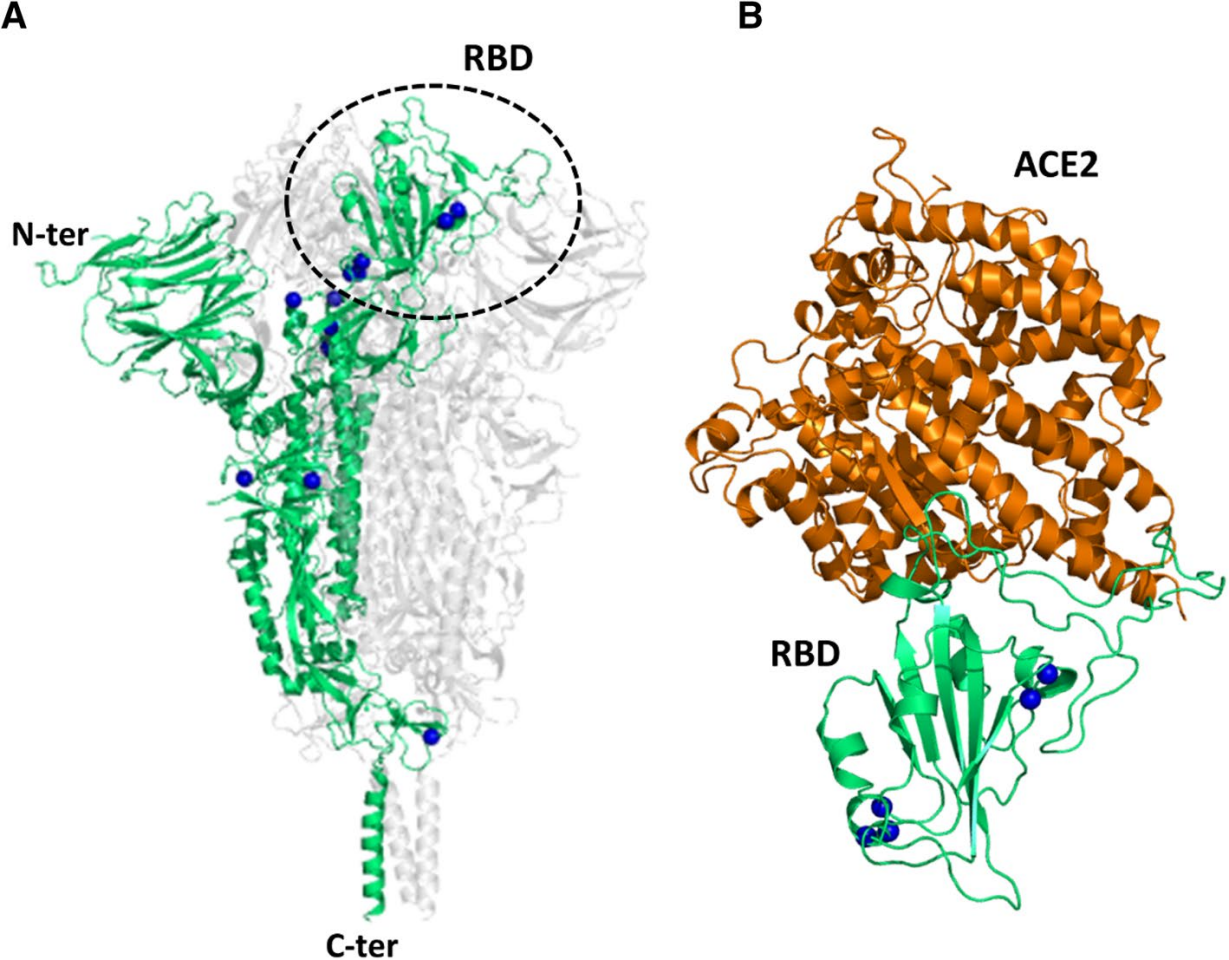
Three-dimensional structure of the SARS-CoV-2 Spike protein. Cartoon representation of (**A**) the protein trimer (PDB ID 6xr8) and (**B**) the complex of the Spike Receptor Binding Domain (RBD) with the cell receptor ACE2 (PDB ID 6m0j). The location of the residues that have never been found to be changed in the DataFeb21 dataset is shown as blue balls.

The data collected considering the mutations at March and October 2020 were compared to those obtained by performing similar analyses on the ensemble of the mutations detected up to 2021 February 7th (DataFeb21—Supplementary Table S3) that essentially corresponds to the first year of the worldwide SARS-CoV-2 spread. This dataset contains 38,986 AA substitutions identified from the analysis of 415,516 genomes. An idea of the mutations accumulated up to 2021 February is provided by the analysis of the replaced residues in the Spike protein, a crucial factor for the virus entry in the host cells and an important target for preventive and therapeutic approaches. Overall, 5809 mutations were found for this protein. Considering that Spike contains 1273 residues, the average mutation occurrences *per* residue is 4.6. The most mutated AA residue is Asp80 that is replaced by 12 other residues out of the 19 possible substitutions. Only 12 residues (Ser383, Lys386, Leu387, Asn422, Tyr423, Gly601, Gln644, Cys749, Arg983, Glu988, Gln992, and Cys1126) of the protein (0.94%) were never mutated. As shown in Fig. 8, all of the residues belonging to the N-terminal domain of the protein were found to be mutated at least once. In this domain, residues presenting the highest frequency of mutation are present (Supplementary Figs. S3 and S4).

**Figure 9:**
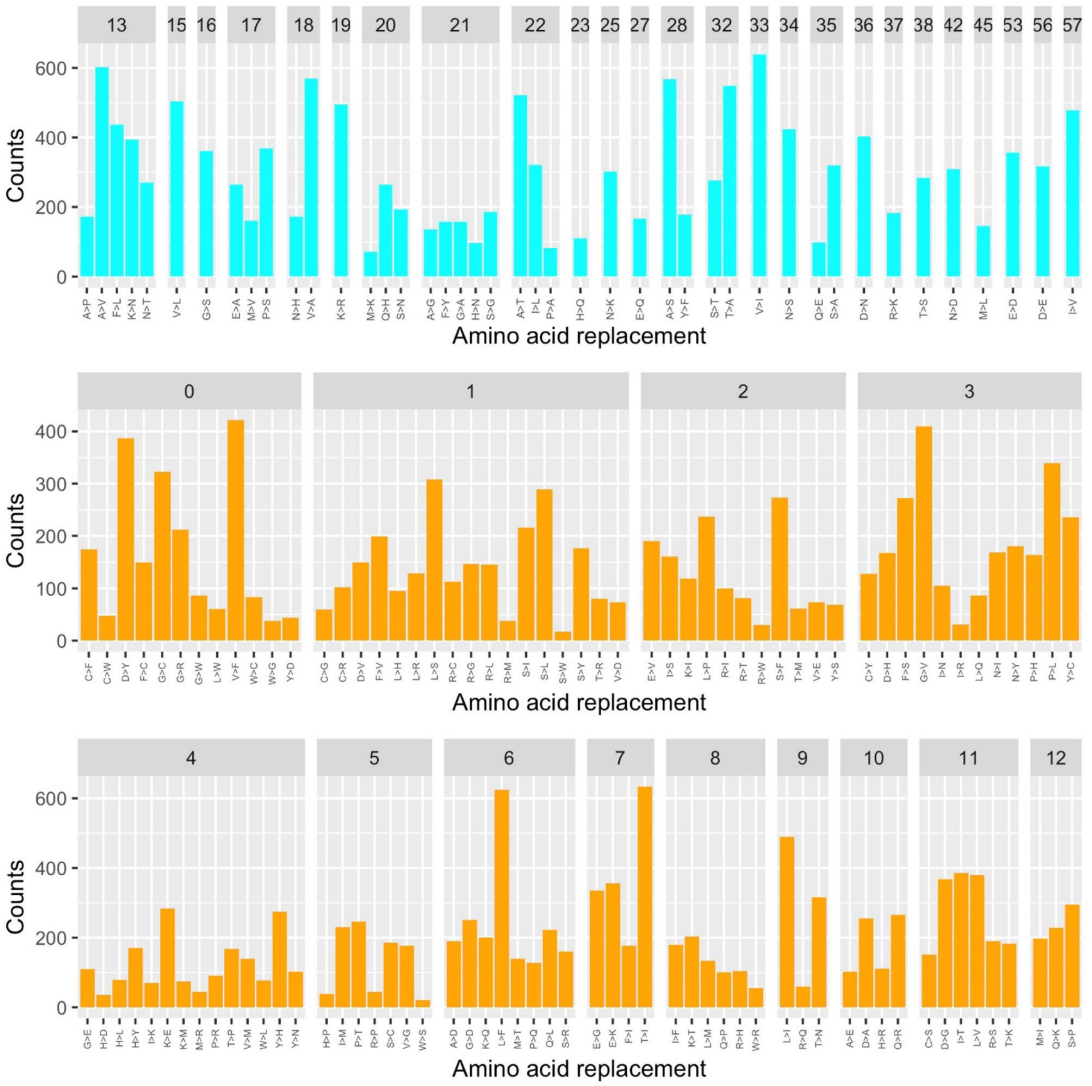
Frequencies of the observed mutations that can occur with a single base change grouped in conservative (cyan) and non-conservative (orange) detected in the DataFeb21 dataset.

**Figure 10:**
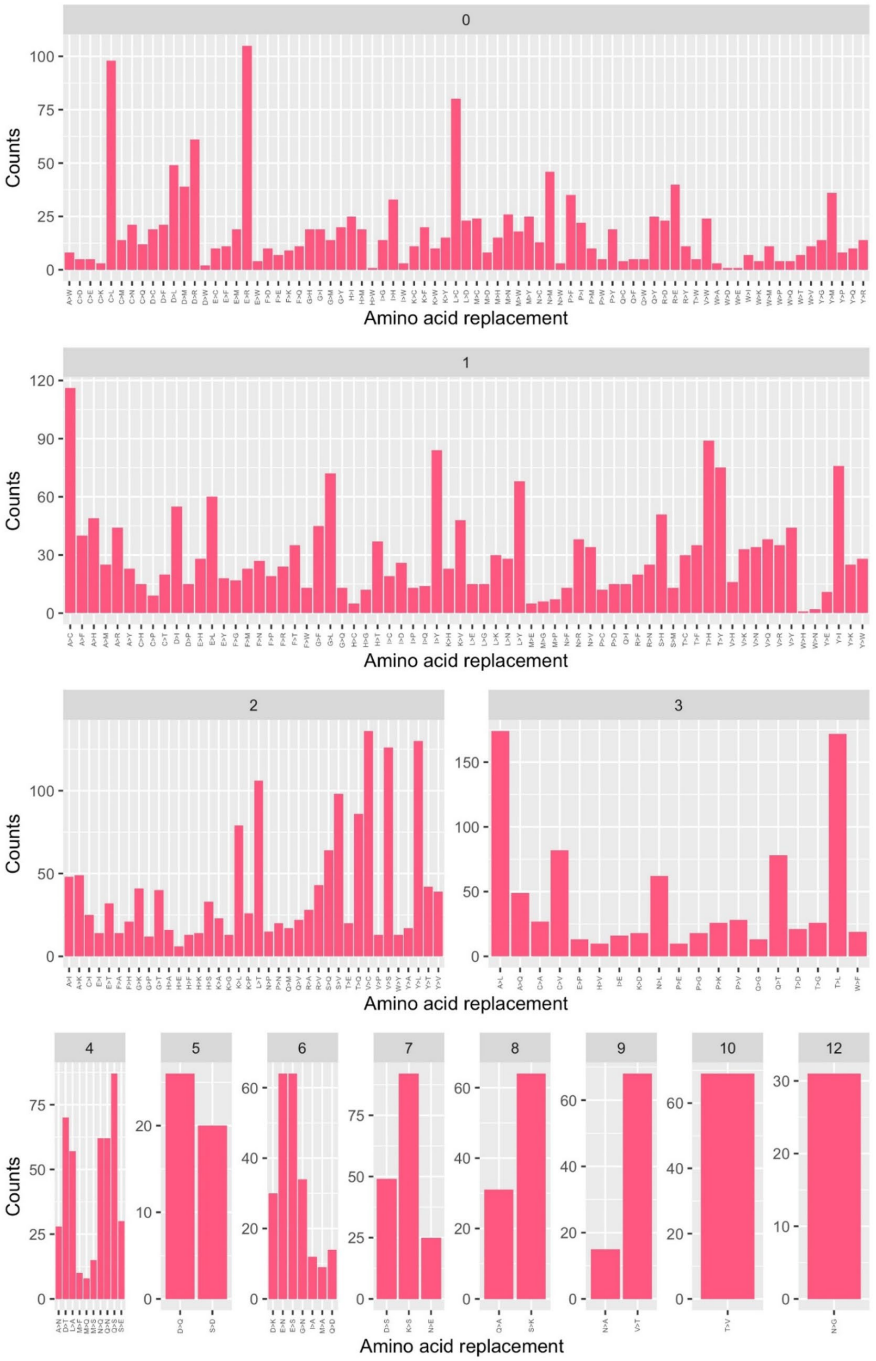
Frequencies of the observed mutations that require more than one base change detected in the DataFeb21 dataset.

All theoretically possible AA substitutions (380) are present in DataFeb21 (Table 1). As found for the earlier datasets, mutations that can occur through a single base change present frequencies that are much higher than those requiring multiple base changes (Figs. 9 and 10). The analysis of the frequencies of these 380 possible substitutions as function of the PAM values and of the number of changes required clearly indicates that conservative mutations are more frequent compared to non-conservative ones (Fig. 9).

**Figure 11:**
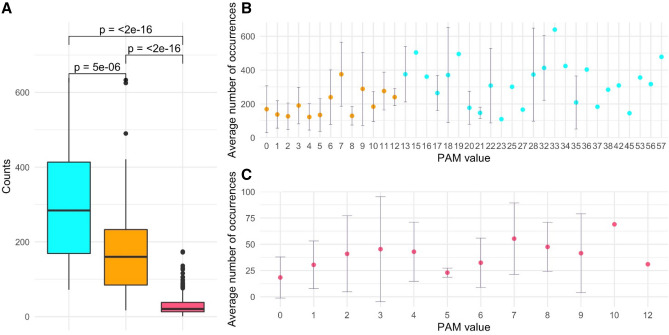
(**A**) Boxplot of the number of occurrences *per* substitution types stratified in conservative (cyan) and non-conservative (orange) types that can occur with a single base change and types that require more than one change (magenta). Average values with standard deviation (bars) of the number of occurrences within PAM values detected in the DataFeb21 dataset: (**B**) substitutions that can occur with one base change grouped in non-conservative (orange) and conservative (cyan) and (**C**) substitutions requiring more than 1 base change (magenta).

For mutations occurring with a single base change, the quantitative comparison of the frequencies of the conservative and non-conservative mutations (Fig. 11A) using the Wilcox-test provides a p-value of 5.0 × 10^–6^. Again, the mutations requiring more than one base change, which are all non-conservative, show considerably lower frequencies compared to the non-conservative mutations occurring in a single base change (Fig. 11A, Wilcox-test p-value < 2 × 10^–16^).

We then checked whether the trends emerged from the global analysis of the mutations could also be detected for the individual proteins of the virus. To this aim, we considered the proteins exhibiting the largest number of mutations (NSP3, Spike, NSP2, NSP12, and N) (Supplementary Table S10). Interestingly, for all of these proteins conservative mutations present frequencies that are significantly higher than those shown by non-conservative ones, thus confirming the trends highlighted by the overall analysis (Supplementary Figs. S5–S9).

When we consider the average occurrences of mutations *per* PAM value, we observe that conservative substitutions generally exhibit larger frequencies than non-conservative ones. As previously observed for DatOct20, also in this case for some PAM values we observe large standard deviations that may be ascribed to the presence of outliers (Fig. 11B,C).

Similarly to what observed in DataOct20, also in DataFeb21 the most frequent replacements are V > I (639 times), T > I (633 times), L > F (625 times) and A > V (602 times) (Fig. 9 and Supplementary Table S5). As observed above, two of these highly occurring mutations (T > I PAM = 7 and L > F PAM = 6) are non-conservative substitutions. The inspection of the occurrences of mutations requiring more than one base change indicates that, as observed in DataOct20, A > L (174 times) and T > L (172 times) are the most frequent ones (Fig. 10). Collectively, these findings indicate that most frequent substitutions led to an increase of the hydrophobicity, independently of the number of codon base changes required for the mutation.

The analysis performed individually on the five most mutated proteins (NSP3, Spike, NSP2, NSP12, and N) indicates analogies and differences among them (Supplementary Table S11). It is interesting to note that the most frequent AA substitutions (V > I, T > I, L > F, A > V, and V > A) detected in DataOct20 and DataFeb21 datasets are among the fifteen most frequent ones also for these proteins with the exception of the protein N. The protein NSP2 shows a high frequency of substitutions causing a decrease of Glu residues whereas a depletion of Gln residues is evident for the protein N (Supplementary Table S11). The analysis of enrichment/depletion in counts of mutated versus original amino acid residues confirms the trends observed for the previous datasets with a significant increase in the two most hydrophobic residues, F and I, and a decrease of hydrophilic residues (Fig. 4C). In addition, the distribution of the ΔHydrophobicity values is slightly shifted toward an increased hydrophobicity (mean ΔHydrophobicity 0.06 ± 1.18) (Fig. 4F). The analysis of the ΔHydrophobicity performed on the individual viral proteins confirms a non-uniform growth of hydrophobicity with the proteins NSP3 and N exhibiting the lowest and the highest values, respectively (Supplementary Fig. S10 and Table S8).

## Discussion

Proteins are fundamental biomolecules that combine remarkable molecular and structural complexity with fine regulation. Although they are made of thousands of atoms, their functional properties may be heavily affected even by the replacement of a handful number of them. In general, missense mutations may lead to radically different consequences in protein structure/function ranging from negligible to dramatic effects. Frequently, they fine-tune protein functions. The a priori prediction of the effect of mutations on the protein function/structure and on their interactome is not an easy task. In this scenario, viruses deserve special attention as they exploit extensive mutations as an adaptive mechanism to the host^10^.

Here we present a global analysis of the AA mutations that have been progressively detected in different sites of the SARS-CoV-2 proteins. This was done by collecting mutations at different stages of the pandemic. We set up three distinct checkpoints: (i) at 2020 March 15th, 2 months after the deposition of the first SARS-CoV-2 genome sequence, (ii) at 2020 October 7th, the early stage of the pandemic spread in the Western countries, and (iii) at 2021 February 7th, essentially one year after the outbreak of the pandemic at global scale.

Mutations were classified according to the similarity of the underlying codons^14^ and to the probability to be detected in highly similar protein sequences (PAM values, Supplementary Table S4)^13^. In particular, the correlation between these two parameters allowed us to discriminate between conservative and non-conservative mutations (Fig. 1).

The analysis of the evolution of SARS-CoV-2 mutations provides some interesting observations. Indeed, mutation types detected at 2020 March 15th (116 out of 380) essentially represent a sub-set (107 out of 150) of the AA substitutions that require a single base change (Table 1). A significant number of the possible single base substitutions (43 out of 150), generally presenting low PAM values, are still missing in the database generated at the first checkpoint (Fig. 2B). The scenario is radically different in the mutation dataset collected at 2020 October 7th where essentially all types of mutations (378 out 380), including those requiring multiple base changes, are observed (Table 1). As expected, the number of mutations and their frequencies further increased in the dataset collected at 2021 February 7th in which all types of substitutions are observed (Table 1).

The analysis of the frequencies of the observed mutation types presents interesting analogies despite the temporal separation of the three checkpoints and the content of the corresponding mutation datasets. In particular, mutations that can occur with a single base change in the codon are by far more frequent than those requiring multiple changes thus indicating that the similarity of the underlying codons is the crucial factor that dictates the occurrence of specific mutations. In all cases, non-conservative mutations, which are characterized by very low PAM values, present rather lower frequencies compared to the conservative ones. This finding may represent a signature of the virus adaptation to humans that is manifested with the elevated frequencies observed for the mutations that do not significantly affect the structure/function of the viral protein. Nevertheless, it is important to note that SARS-CoV-2 genomes are accumulating non-conservative mutations that have very low probabilities to occur in evolutionary-related proteins displaying very high overall identities (99%) as those used to generate the PAM1 matrix.

Despite the huge difference in the number of the mutations of DataMar20 and DataOct20 datasets (404 versus 25,634), they share some of the most frequent substitutions (T > I, L > F, and A > V). It is worth mentioning that these mutations do not present, among single base substitutions, high theoretical probabilities to occur^14^. In general, in both cases, we observe an enrichment of hydrophobic residues associated with the mutation events, in line with previous literature reports^12^. The large content of mutations (38,986) that are present in the DataFeb21 dataset allowed the analysis of the most frequent AA substitutions in the most mutated SARS-CoV-2 viral proteins. Interestingly, although the global trends are also observed in most of these proteins (e.g. NSP3, Spike, NSP2, and NSP12), a specific mutational trend is exhibited by the N protein in which a depletion of Gln residues is evident. An increase of the hydrophobicity has also been detected at the individual protein level for most of the SARS-CoV-2 proteins.

The analysis of the diffusion of specific missense mutations in the human population has received particular attention throughout the pandemic evolution^3,8,9,12–22^. It has been pointed out that the D614G mutation, a non-conservative mutation with a PAM value of 11, in the Spike protein, which occurred through a single base change, has increased the virus infectivity^23–25^. More recently, other mutations of the Spike protein have been reported to be crucial for the virus infectivity of other variants (“Emerging SARS-CoV-2 Variants”. Centers for Disease Control and Prevention. https://www.cdc.gov/). Most of these mutations (K417T, L452R, T478K, E484K, N501Y, H655Y, P681H, and P681R) are non-conservative as they exhibit PAM values in the interval 1–11. Two of them (K417N and A701V) are barely conservative as they present a PAM value of 13. It is important to note that, despite the relative abundance of AA substitution types with PAM values of 0 (89 out of 380), none of them is present the Spike mutants of these variants. Collectively, these observations indicate that the virus has acquired an increased infectivity through non-conservative but not radical mutations. In this scenario, we believe that the monitoring of the diffusion of the non-conservative mutations here identified and classified, which may underlie significant structural/functional changes, could highlight widespread SARS-CoV-2 variants with altered properties.

In conclusion, the present study provides interesting indications of the early evolution of the virus and useful tools for the global and genome-specific evaluation of the impact that mutations could have on the structure/function of SARS-CoV-2 variants that emerged or will emerge in the pandemic. The unique availability of genome data since the early stage of the pandemic has provided information about the first AA substitutions occurring in the viral proteins. Notably, the most frequent mutations have remained essentially the same over one year of the pandemic.

## Methods

### Source of the data

The lists of the AA mutations present in SARS-CoV-2 variants detected using the sequence of the Wuhan genome (GISAID accession ID: EPI_ISL_402124) as reference were retrieved from the Global Initiative for Sharing All Influenza Data (GISAID) database (https://www.gisaid.org/)^1^ at three time points: 2020 March 15th (DataMar20—Supplementary Table S1), 2020 October 7th (DataOct20—Supplementary Table S2) and 2021 February 7th (DataFeb21—Supplementary Table S3). In particular, for each viral protein, we retrieved from the server the mutations that were manually curated to eliminate non-missense mutations. These were then merged to carry out the global analyses.

### Classification of the mutations

The AA substitutions were classified according to two criteria. First, we considered the Point Accepted Mutation (PAM) score (Supplementary Table S4), i.e. the likelihood that an AA substitution is accepted by the natural selection^13^. Second, we considered if the AA substitution required one or more than one base change in the genetic code to happen^14^.

Differences in hydrophobicity (ΔHydrophobicity) between the mutated and the original residue was calculated based on the consensus hydrophobic scale developed by Eisenberg^26^ and reported in Supplementary Table S6.

We introduced the genome divergence index (GDI) to measure the divergence of a genome from the reference sequence (GISAID accession ID: EPI_ISL_402124). For each polypeptide chain encoded by a specific SARS-CoV-2 genome, the GDI is calculated as GDI = Σ_i_ (58-PAM1_i_), where PAM1_i_ is the PAM1 score of the i-th AA substitution in the polypeptide chain, and 58 was chosen to have positive values of the score considering that PAM1i values range from 1 to 57. In this way, the most conservative mutation (I > V, PAM = 57) has value 1 in the summation. Statistical analyses were performed using R (R Core Team (2020). R: A language and environment for statistical computing. R Foundation for Statistical Computing, Vienna, Austria. URL https://www.R-project.org/).

## Supplementary Information

Supplementary Information.

Supplementary Table S1.

Supplementary Table S2.

Supplementary Table S3.
