# Supplementary material for: A global analysis of conservative and non-conservative mutations in SARS-CoV-2 detected in the first year of the COVID-19 world-wide diffusion
Source: Sci Rep. 2021 Dec 30;11:24495. doi: 10.1038/s41598-021-04147-1 (PMC8718531; doi:10.1038/s41598-021-04147-1)
Supplement: Supplementary file 1 — Supplementary Information. [file 41598_2021_4147_MOESM1_ESM.pdf]

Supplementary Material

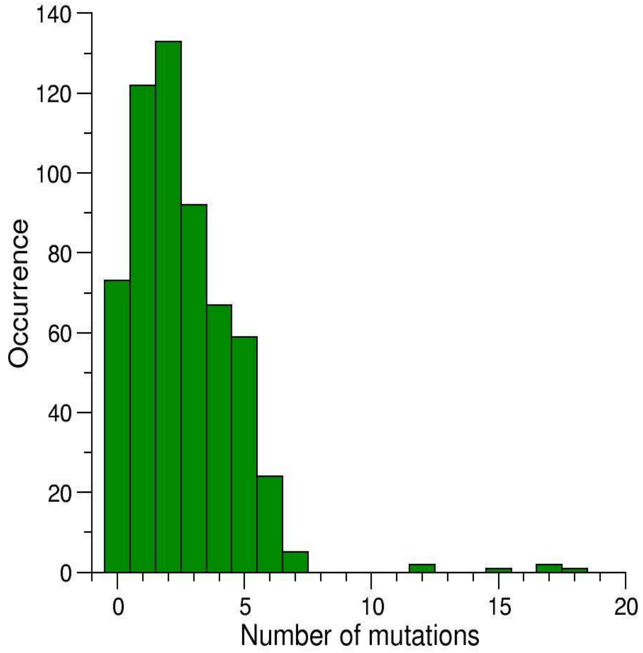

Supplementary Figure S1. Number of amino acid mutations *per* genome. The analysis has been conducted on the 581 SARS-CoV-2 genomes deposited in the GISAID database up to 2020 March 15th using the sequence of the Wuhan genome as reference (GISAID accession ID: EPI_ISL_402124).

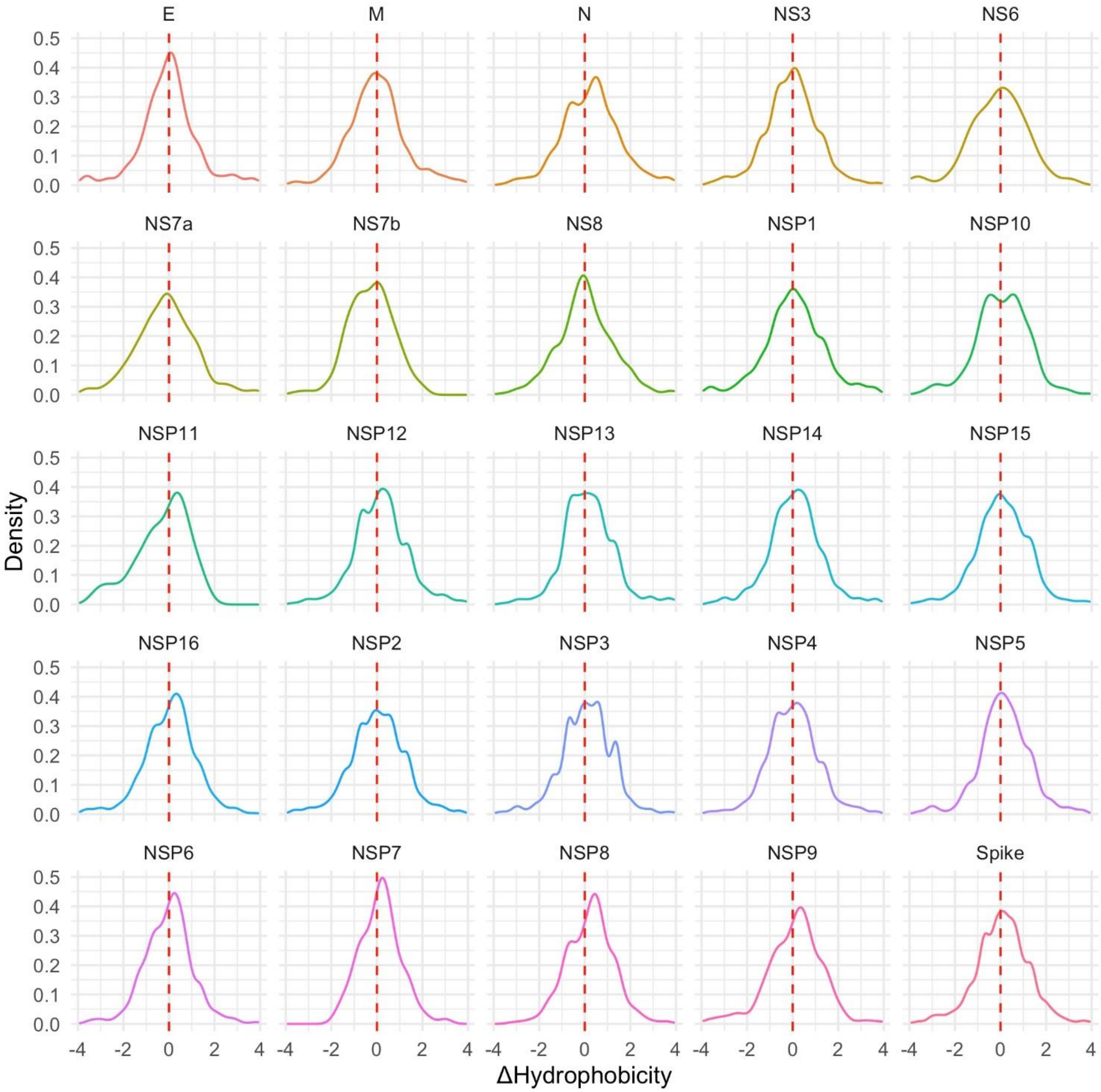

Supplementary Figure S2. Density plots of the ΔHydrophobicity values associated to the AA replacements detected in the individual SARS-CoV-2 proteins of the DataOct20 dataset.

```
123456789012345678901234567890123456789012345678901234567890
MFVFLVLLPLVSSQCVNLTTRTQLPPAYTNSFTRGVYYPDKVFRSSVLHSTQDLFLPFFS   60
NVTWFHAIHVSGTNGTKRFDNPVLPFNDGVYFASTEKSNIIRGWIFGTTLDSKTQSLLIV  120
NNATNVVIKVCEFQFCNDPFLGVYYHKNNKSWMESEFRVYSSANNCTFEYVSQPFLMDLE  180
GKQGNFKNLREFVFKNIDGYFKIYSKHTPINLVRDLPQGFSALEPLVDLPIGINITRFQT  240
LLALHRSYLTPGDSSSGWTAGAAAYYVGYLQPRTFLLKYNENGTITDAVDCALDPLSETK  300
CTLKSFTVEKGIYQTSNFRVQPTESIVRFPNITNLCPFGEVFNATRFASVYAWNRKRISN  360
CVADYSVLYNSASFSTFKCYGVSPTKLNDLCFTNVYADSFVIRGDEVRQIAPGQTGKIAD  420
YNYKLPDDFTGCVIAWNSNNLDSKVGGNYNYLYRLFRKSNLKPFERDISTEIYQAGSTPC  480
NGVEGFNCYFPLQSYGFQPTNGVGYQPYRVVVLSFELLHAPATVCGPKKSTNLVKNKCVN  540
FNFNGLTGTGVLTESNKKFLPFQQFGRDIADTTDAVRDPQTLEILDITPCSFGGVSVITP  600
GTNTSNQVAVLYQDVNCTEVPVAIHADQLTPTWRVYSTGSNVFQTRAGCLIGAEHVNNSY  660
ECDIPIGAGICASYQTQTNSPRRARSVASQSIIAYTMSLGAENSVAYSNNSIAIPTNFTI  720
SVTTEILPVSMTKTSVDCTMYICGDSTECSNLLLQYGSFCTQLNRALTGIAVEQDKNTQE  780
VFAQVKQIYKTPPIKDFGGFNFSQILPDPSKPSKRSFIEDLLFNKVTLADAGFIKQYGDC  840
LGDIAARDLICAQKFNGLTVLPPLLTDEMIAQYTSALLAGTITSGWTFGAGAALQIPFAM  900
QMAYRFNGIGVTQNVLYENQKLIANQFNSAIGKIQDSLSSTASALGKLQDVVNQNAQALN  960
TLVKQLSSNFGAISSVLNDILSRLDKVEAEVQIDRLITGRLQSLQTYVTQQLIRAAEIRA 1020
SANLAATKMSECVLGQSKRVDFCGKGYHLMSFPQSAPHGVVFLHVTYVPAQEKNFTTAPA 1080
ICHDGKAHFPREGVFVSNGTHWFVTQRNFYEPQIITTDNTFVSGNCDVVIGIVNNTVYDP 1140
LQPELDSFKEELDKYFKNHTSPDVDLGDISGINASVVNIQKEIDRLNEVAKNLNESLIDL 1200
QELGKYEQYIKWPWYIWLGFIAGLIAIVMVTIMLCCMTSCCSCLKGCCSCGSCCKFDEDD 1260
SEPVLKGVKLHYT
```

Supplementary Figure S3. Amino acid sequence of the SARS-CoV-2 Spike protein (UNIPROT code P0DTC2). Residues that have never been found to be replaced and those found to be changed more than seven times in the dataset DataFeb21 are highlighted in cyan and red, respectively.

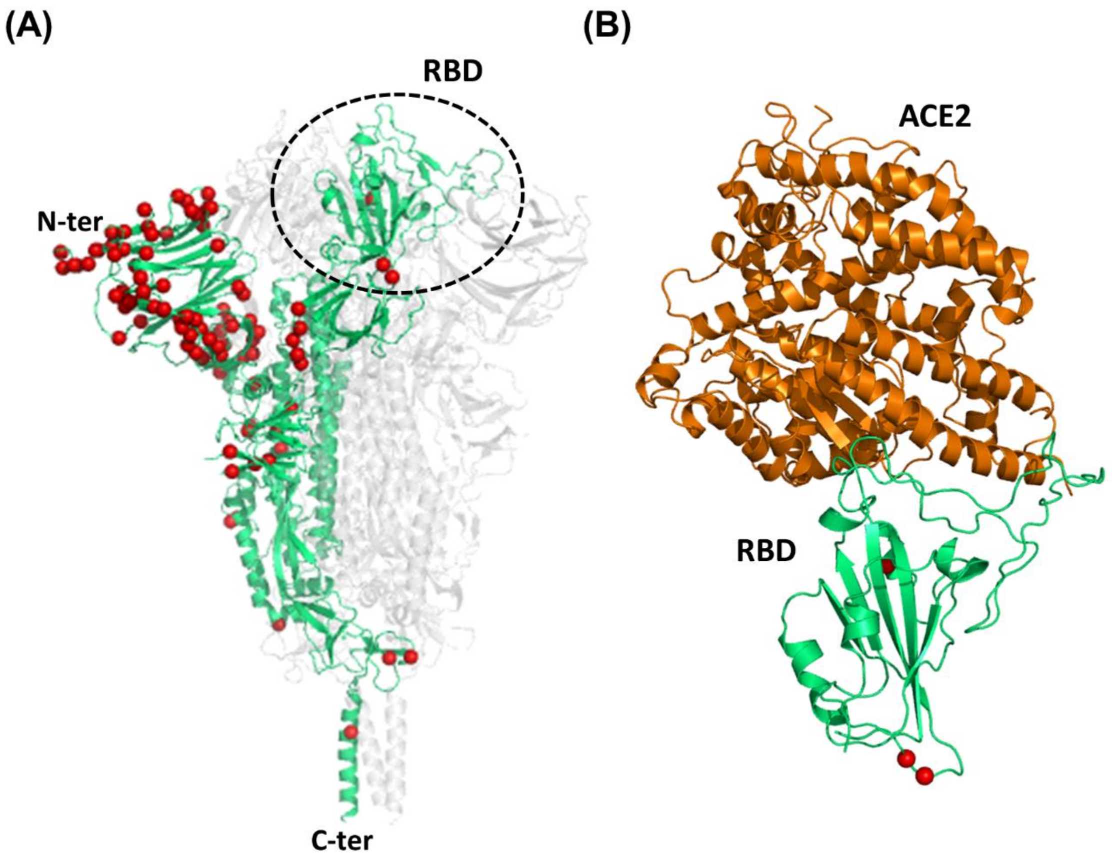

Supplementary Figure S4. Three-dimensional structure of the SARS-CoV-2 Spike protein. Cartoon representation of **(A)** the protein trimer (PDB ID 6xr8) and **(B)** the complex of the Spike Receptor Binding Domain (RBD) with the cell receptor ACE2 (PDB ID 6m0j). The location of the residues that have been found to be changed more than seven times in the DataFeb21 dataset is shown as red balls.

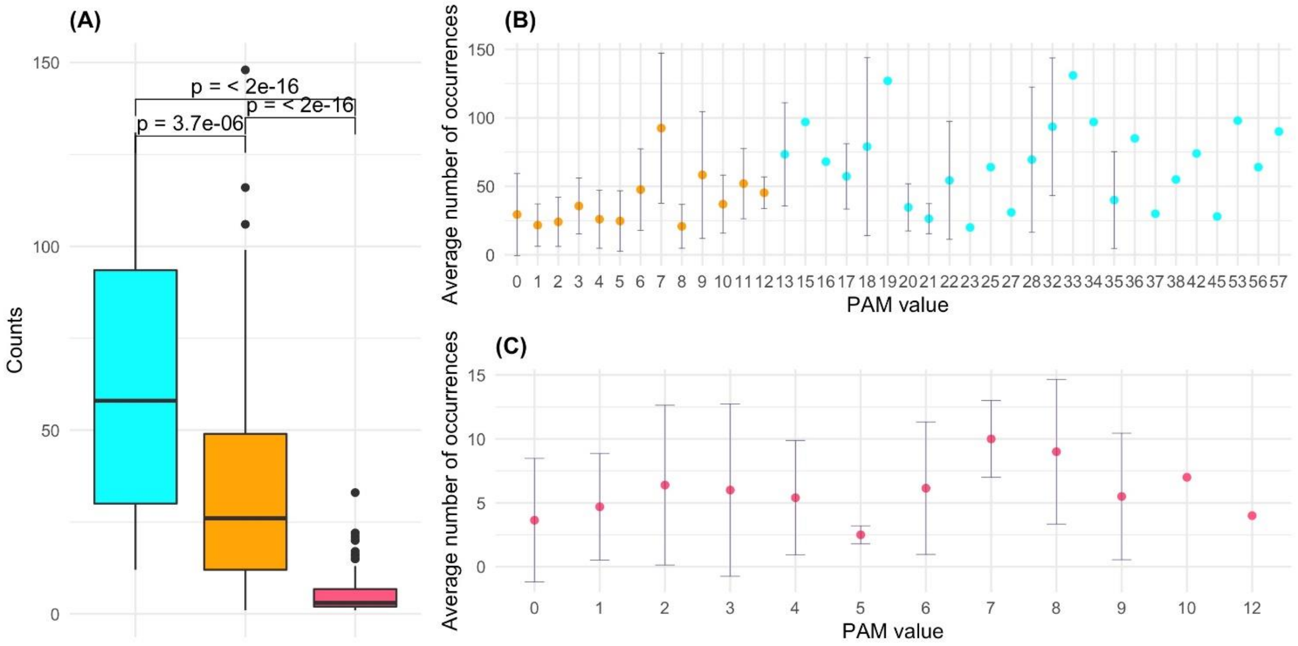

Supplementary Figure S5. (A) Boxplot of the number of occurrences *per* substitution types stratified in conservative (cyan) and non-conservative (orange) types that can occur with a single base change and types that require more than one change (magenta) detected for NSP3 protein in the DataFeb21 dataset. Average values with standard deviation (bars) of the number of occurrences within PAM values detected for NSP3 in the DataFeb21 dataset: **(B)** substitutions that can occur with one base change grouped in non-conservative (orange) and conservative (cyan) and **(C)** substitutions requiring more than 1 base change (magenta).

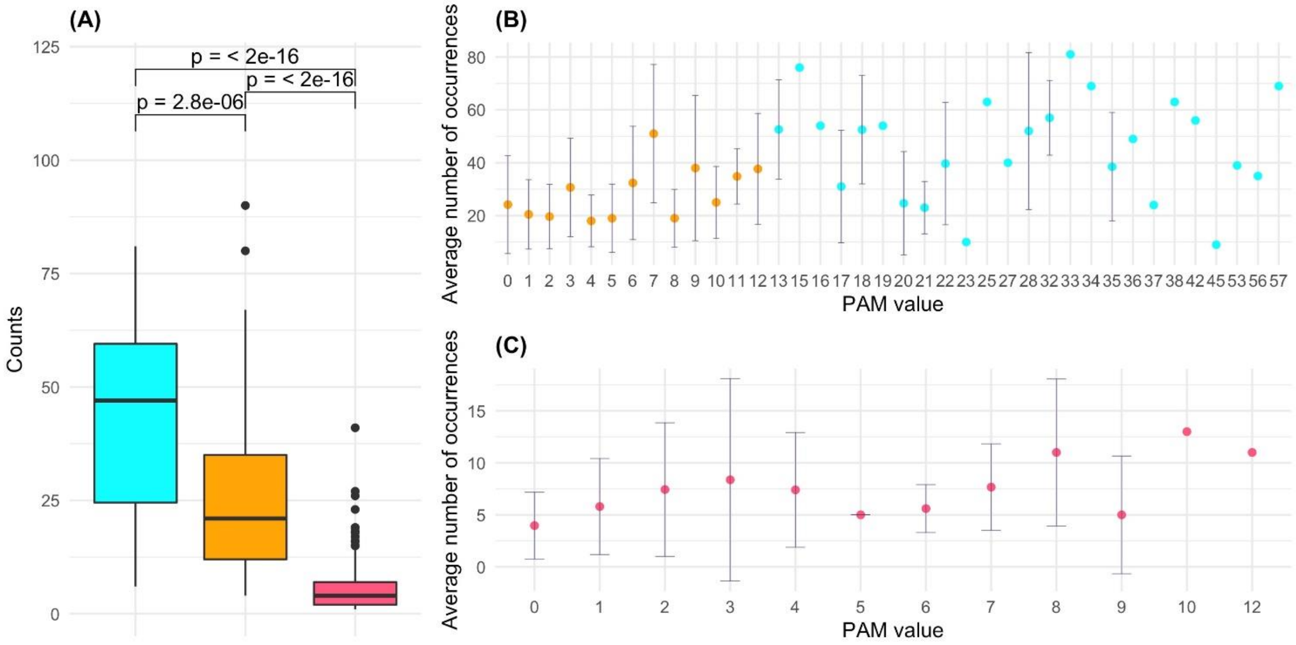

Supplementary Figure S6. (A) Boxplot of the number of occurrences *per* substitution types stratified in conservative (cyan) and non-conservative (orange) types that can occur with a single base change and types that require more than one change (magenta) detected for Spike protein in the DataFeb21 dataset. Average values with standard deviation (bars) of the number of occurrences within PAM values detected for Spike in the DataFeb21 dataset: **(B)** substitutions that can occur with one base change grouped in non-conservative (orange) and conservative (cyan) and **(C)** substitutions requiring more than 1 base change (magenta).

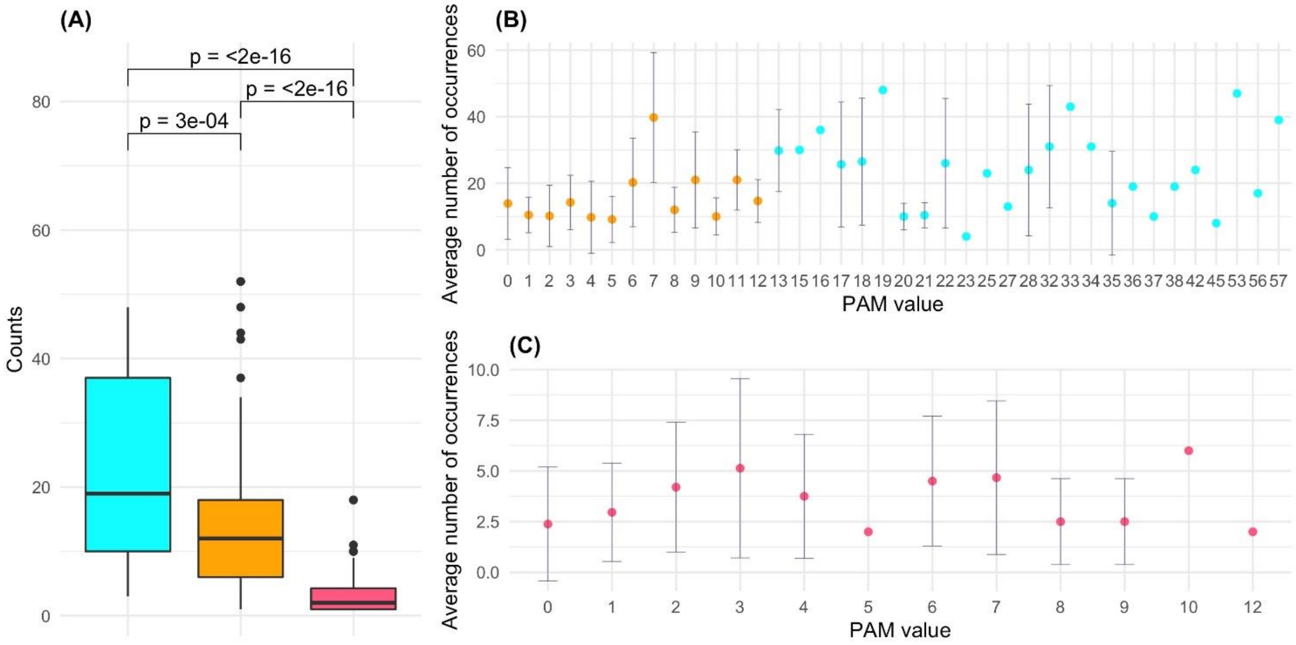

Supplementary Figure S7. (A) Boxplot of the number of occurrences *per* substitution types stratified in conservative (cyan) and non-conservative (orange) types that can occur with a single base change and types that require more than one change (magenta) detected for NSP2 protein in the DataFeb21 dataset. Average values with standard deviation (bars) of the number of occurrences within PAM values detected for NSP2 in the DataFeb21 dataset: **(B)** substitutions that can occur with one base change grouped in non-conservative (orange) and conservative (cyan) and **(C)** substitutions requiring more than 1 base change (magenta).

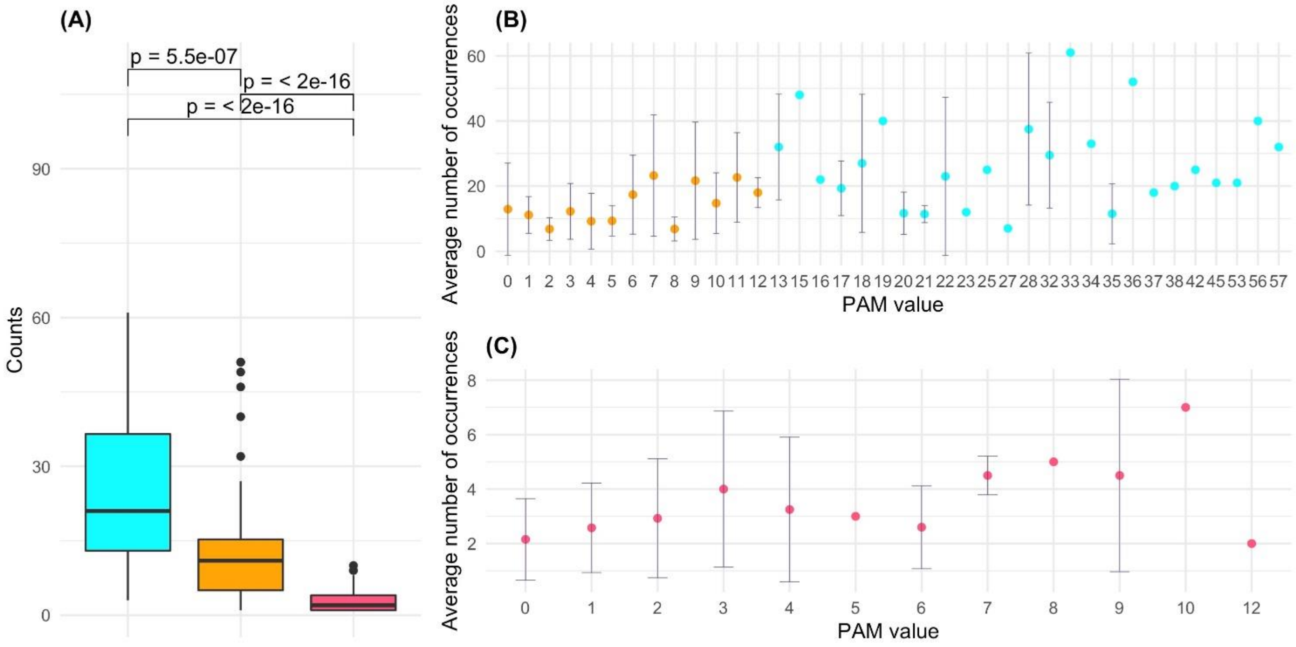

Supplementary Figure S8. (A) Boxplot of the number of occurrences *per* substitution types stratified in conservative (cyan) and non-conservative (orange) types that can occur with a single base change and types that require more than one change (magenta) detected for NSP12 protein in the DataFeb21 dataset. Average values with standard deviation (bars) of the number of occurrences within PAM values detected for NSP12 in the DataFeb21 dataset: **(B)** substitutions that can occur with one base change grouped in non-conservative (orange) and conservative (cyan) and **(C)** substitutions requiring more than 1 base change (magenta).

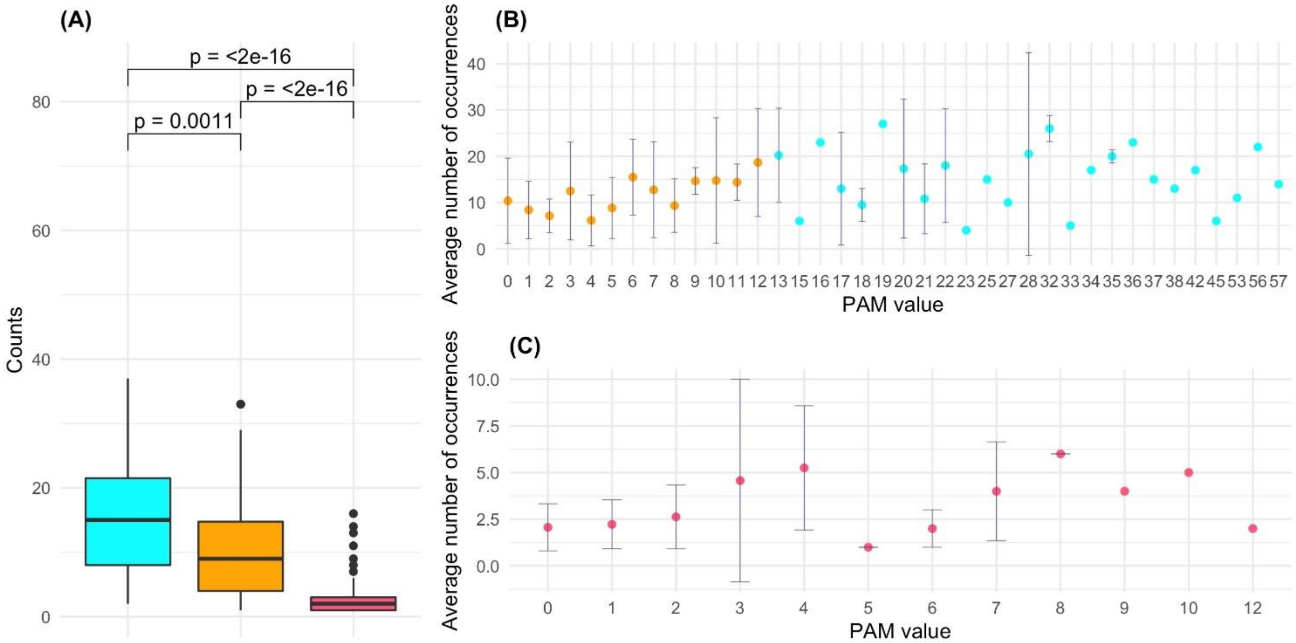

Supplementary Figure S9. (**A**) Boxplot of the number of occurrences *per* substitution types stratified in conservative (cyan) and non-conservative (orange) types that can occur with a single base change and types that require more than one change (magenta) detected for N protein in the DataFeb21 dataset. Average values with standard deviation (bars) of the number of occurrences within PAM values detected for N in the DataFeb21 dataset: **(B)** substitutions that can occur with one base change grouped in non-conservative (orange) and conservative (cyan) and **(C)** substitutions requiring more than 1 base change (magenta).

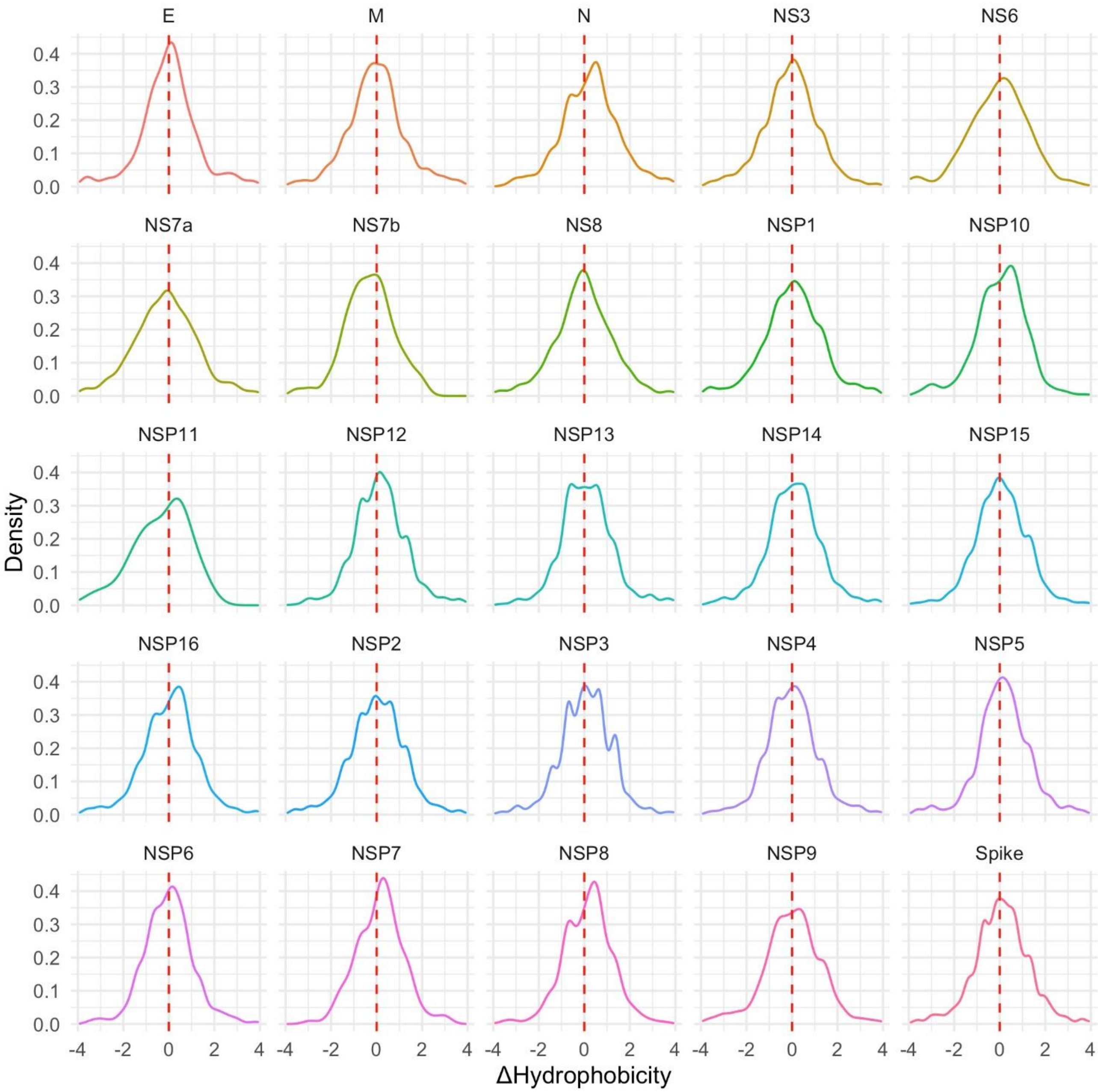

Supplementary Figure S10. Density plots of the ΔHydrophobicity values associated to the AA replacements detected in the individual SARS-CoV-2 proteins of the DataFeb21 dataset.

Supplementary Table S1. Ensemble of 404 amino acid mutations (DataMar20) derived from 581 sequences of SARS-CoV-2 variants deposited up to 2020 March 15th. Mutations have been obtained using the Wuhan genome (GISAID accession ID: EPI_ISL_402124) as reference sequence.

Supplementary Table S2. Ensemble of 25,634 amino acid mutations (DataOct20) derived from 135,404 sequences of SARS-CoV-2 variants deposited up to 2020 October 7^{h}. Mutations have been obtained using the Wuhan genome (GISAID accession ID: EPI_ISL_402124) as reference sequence.

Supplementary Table S3. Ensemble of 38,986 amino acid mutations (DataFeb21) derived from 415,516 sequences of SARS-CoV-2 variants deposited up to 2021 February 7^{h}. Mutations have been obtained using the Wuhan genome (GISAID accession ID: EPI_ISL_402124) as reference sequence.

Supplementary Table S4. List of PAM values associated to all the 380 possible amino acid substitution types. The PAM value corresponds to the probability (multiplied by 10,000) that a certain amino acid (original AA) will be replaced by another amino acid (replacement AA) after the evolutionary interval corresponding to 1 accepted point mutation *per* 100 amino acids. In other words, the PAM1 matrix estimates the rate of each substitution that would be expected if 1% of the amino acids had changed. The diagonal corresponds to the probability of having a residue unchanged in the homolog sequences (i.e. it is replaced with itself). Amino acids are colored according to their chemico-physical properties (acidic in light red, basic in light blue, hydrophobic in orange, polar in green, Gly and Pro in violet).

ORIGINAL AMINO ACID

REPLACEMENT AMINO ACID

	A	R	N	D	C	Q	E	G	H	I	L	K	M	F	P	S	T	W	Y	V
**A**	9867	2	9	10	3	8	17	21	2	6	4	2	6	2	22	35	32	0	2	18
**R**	1	9913	1	0	1	10	0	0	10	3	1	19	4	1	4	6	1	8	0	1
**N**	4	1	9822	36	0	4	6	6	21	3	1	13	0	1	2	20	9	1	4	1
**D**	6	0	42	9859	0	6	53	6	4	1	0	3	0	0	1	5	3	0	0	1
**C**	1	1	0	0	9973	0	0	0	1	1	0	0	0	0	1	5	1	0	3	2
**Q**	3	9	4	5	0	9876	27	1	23	1	3	6	4	0	6	2	2	0	0	1
**E**	10	0	7	56	0	35	9865	4	2	3	1	4	1	0	3	4	2	0	1	2
**G**	21	1	12	11	1	3	7	9935	1	0	1	2	1	1	3	21	3	0	0	5
**H**	1	8	18	3	1	20	1	0	9912	0	1	1	0	2	3	1	1	1	4	1
**I**	2	2	3	1	2	1	2	0	0	9872	9	2	12	7	0	1	7	0	1	33
**L**	3	1	3	0	0	6	1	1	4	22	9947	2	45	13	3	1	3	4	2	15
**K**	2	37	25	6	0	12	7	2	2	4	1	9926	20	0	3	8	11	0	1	1
**M**	1	1	0	0	0	2	0	0	0	5	8	4	9874	1	0	1	2	0	0	4
**F**	1	1	1	0	0	0	0	1	2	8	6	0	4	9946	0	2	1	3	28	0
**P**	13	5	2	1	1	8	3	2	5	1	2	2	1	1	9926	12	4	0	0	2
**S**	28	11	34	7	11	4	6	16	2	2	1	7	4	3	17	9840	38	5	2	2
**T**	22	2	13	4	1	3	2	2	1	11	2	8	6	1	5	32	9871	0	2	9
**W**	0	2	0	0	0	0	0	0	0	0	0	0	0	1	0	1	0	9976	1	0
**Y**	1	0	3	0	3	0	1	0	4	1	1	0	0	21	0	1	1	2	9945	1
**V**	13	2	1	1	3	2	2	3	3	57	11	1	17	1	3	2	10	0	2	9901

Supplementary Table S5. List of the fifteen most frequent AA substitution types (AA s.t.) detected in DataMar20, DataOct20, and DataFeb21 datasets.

DataMar20		DataOct20		DataFeb21	
**AA s.t.**	**Frequency**	**AA s.t.**	**Frequency**	**AA s.t.**	**Frequency**
T>I	30	T>I	573	V>I	639
A>V	21	A>V	529	T>I	633
P>S	19	L>F	517	L>F	625
L>F	17	V>I	473	A>V	602
P>L	11	A>S	428	V>A	569
A>S	10	I>V	380	A>S	568
S>L	10	A>T	376	T>A	549
K>N	9	V>L	368	A>T	522
V>L	9	T>A	331	V>L	504
G>S	9	V>F	327	K>R	495
S>F	8	P>S	323	L>I	490
G>V	7	F>L	321	I>V	478
H>Y	7	V>A	320	F>L	437
V>I	7	K>R	311	N>S	424
D>E	7	L>I	296	V>F	421

Supplementary Table S6. Eisenberg scale of the hydrophobicity values for all the 20 natural-encoded amino acids.

**Amino acid**	**Hydrophobicity**
A	0.62
C	0.29
D	-0.9
E	-0.74
F	1.19
G	0.48
H	-0.4
I	1.38
K	-1.5
L	1.06
M	0.64
N	-0.78
P	0.12
Q	-0.85
R	-2.53
S	-0.18
T	-0.05
V	1.08
W	0.81
Y	0.26

Supplementary Table S7. Values of the genome divergence index (GDI) detected for the most mutated SARS-CoV-2 variants deposited in the GISAID database up to 2020 March 15th.

GISAID accession ID	Number of mutations	GDI
EPI_ISL_406592	18	873
EPI_ISL_408483	17	784
EPI_ISL_406799	17	748
EPI_ISL_408487	15	671
EPI_ISL_412900	12	627
EPI_ISL_413485	12	581
EPI_ISL_408485	7	358
EPI_ISL_414562	7	330
EPI_ISL_414560	7	305
EPI_ISL_413588	7	281
EPI_ISL_413570	7	281

Supplementary Table S8. Average values of ΔHydrophobicity detected for the individual SARS-CoV-2 proteins in DataOct20 and DataFeb21 datasets.

**Protein**	**Mean value of ΔHydrophobicity**	
	**DataOct20**	**DataFeb21**
NSP1	0.075	0.047
NSP2	0.039	0.023
NSP3	0.074	0.066
NSP4	0.018	0.0045
NSP5	0.17	0.15
NSP6	-0.024	-0.011
NSP7	0.15	0.15
NSP8	0.23	0.15
NSP9	0.095	0.029
NSP10	0.058	0.029
NSP11	-0.29	-0.33
NSP12	0.17	0.15
NSP13	0.16	0.12
NSP14	0.10	0.096
NSP15	0.087	0.057
NSP16	0.045	0.034
Spike	0.082	0.082
NS3	-0.047	-0.069
E	-0.015	0.0018
M	0.014	-0.021
NS6	-0.077	-0.070
NS7a	-0.14	-0.15
NS7b	-0.27	-0.31
NS8	0.039	-0.028
N	0.27	0.26

Supplementary Table S9. Values of the genome divergence index (GDI) detected for the most mutated SARS-CoV-2 variants deposited in the GISAID database up to 2020 October 7th.

GISAID accession ID	Number of mutations	GDI
EPI_ISL_547877	215	9551
EPI_ISL_408487	131	6737
EPI_ISL_413696	121	5595
EPI_ISL_435059	107	5101
EPI_ISL_427289	102	5320
EPI_ISL_413695	101	4964
EPI_ISL_483719	90	4743
EPI_ISL_522406	75	3383
EPI_ISL_427291	70	3594
EPI_ISL_507007	68	3353
EPI_ISL_565918	60	3129
EPI_ISL_478055	58	3017
EPI_ISL_483706	58	3096
EPI_ISL_456436	57	2972
EPI_ISL_513011	56	2697
EPI_ISL_437536	55	2482
EPI_ISL_433275	53	2781
EPI_ISL_476839	53	2778
EPI_ISL_483723	53	2783
EPI_ISL_419427	51	2740
EPI_ISL_565909	49	2537
EPI_ISL_424731	48	2538
EPI_ISL_426880	48	2380
EPI_ISL_480330	48	2429
EPI_ISL_478110	47	2466
EPI_ISL_500443	47	2471
EPI_ISL_419398	46	2458
EPI_ISL_509414	46	2417
EPI_ISL_437823	45	2204
EPI_ISL_419858	44	2179
EPI_ISL_431781	43	2311
EPI_ISL_415618	41	1973
EPI_ISL_509412	40	2130
EPI_ISL_494763	39	1866
EPI_ISL_525470	39	1891
EPI_ISL_431292	38	1942
EPI_ISL_500415	38	2002
EPI_ISL_521462	38	1992

EPI_ISL_448303	37	1916
EPI_ISL_456437	37	1982
EPI_ISL_412900	36	1878
EPI_ISL_459244	36	1958
EPI_ISL_480583	36	1853
EPI_ISL_565913	36	1875
EPI_ISL_413747	34	1606
EPI_ISL_419442	34	1723
EPI_ISL_450557	34	1786
EPI_ISL_480611	34	1801
EPI_ISL_519411	32	1705
EPI_ISL_435623	31	1448
EPI_ISL_450926	31	1414
EPI_ISL_480692	31	1538
EPI_ISL_516769	31	1731
EPI_ISL_419429	30	1499
EPI_ISL_457410	30	1652
EPI_ISL_475734	30	1612
EPI_ISL_479425	30	1659
EPI_ISL_479770	30	1625
EPI_ISL_493894	30	1643
EPI_ISL_565910	30	1590

Supplementary Table S10. Number of AA substitutions detected for the individual SARS-CoV-2 proteins in DataFeb21 dataset.

**Protein**	**AA substitutions**
NSP3	7257
Spike	5809
NSP2	3012
NSP12	2780
N	2038
NSP14	1984
NSP4	1810
NSP13	1783
NS3	1611
NSP15	1421
NSP16	1105
NSP6	1090
NSP5	1042
NSP1	1040
NS7a	950
M	773
NS8	737
NSP8	593
NSP10	540
NS6	360
E	315
NS7b	298
NSP9	295
NSP7	279

Supplementary Table S11. List of the fifteen most frequent AA substitution types (AA s.t.) detected for the five most mutated SARS-CoV-2 proteins (NSP3, Spike, NSP2, NSP12, and N) in DataFeb21 dataset. The five most frequent AA substitution types (V>I, T>I, L>F, A>V, and V>A) detected in DataOct20 and DataFeb21 datasets are highlighted in yellow.

**NSP3**		**SPIKE**		**NSP2**		**NSP12**		**N**	
**AA s.t.**	**Freq.**	**AA s.t.**	**Freq.**	**AA s.t.**	**Freq.**	**AA s.t.**	**Freq.**	**AA s.t.**	**Freq.**
T>I	148	T>I	90	E>G	52	V>I	61	A>V	37
V>I	131	V>I	81	E>K	52	A>S	54	A>S	36
T>A	129	L>F	80	L>F	48	D>N	52	G>V	33
K>R	127	V>L	76	K>R	48	D>Y	51	Q>L	33
V>A	125	F>L	73	E>D	47	T>I	51	Q>R	33
A>V	121	A>S	73	E>A	46	A>V	51	Q>H	32
L>F	116	N>S	69	T>I	44	A>T	50	A>T	32
A>S	107	I>V	69	T>A	44	D>G	49	Q>K	29
E>K	106	G>V	67	K>E	43	V>L	48	T>I	28
E>G	99	A>V	67	A>V	43	L>F	46	T>A	28
K>N	99	V>A	67	V>I	43	F>L	46	P>S	27
A>T	98	T>A	67	A>T	41	V>A	42	K>R	27
E>D	98	V>F	64	K>N	40	T>A	41	P>L	26
L>I	97	N>K	63	V>A	40	L>I	40	S>T	24
V>L	97	T>S	63	I>V	39	K>R	40	G>S	23
